# Characterization of Ingredients Incorporated in the Traditional Mixed-Salad of the Capuchin Monks

**DOI:** 10.3390/plants11030301

**Published:** 2022-01-24

**Authors:** Laura Cornara, Gabriele Ambu, Alex Alberto, Domenico Trombetta, Antonella Smeriglio

**Affiliations:** 1 Department of Earth, Environment and Life Sciences, University of Genova, C.so Europa 26, 16132 Genova, Italy; gabrieleambu1976@gmail.com (G.A.); alex.universita.12@gmail.com (A.A.); 2 Department of Chemical, Biological, Pharmaceutical and Environmental Sciences, University of Messina, Viale Ferdinando Stagno d’Alcontres 31, 98166 Messina, Italy; dtrombetta@unime.it

**Keywords:** human diet, edible wild plants, *Plantago coronopus* L., *Rumex acetosa* L., *Cichorium intybus* L., *Artemisia dracunculus* L., phytochemistry, antioxidant activity, anti-inflammatory properties

## Abstract

Recipes on the composition of the “salad of the monks” (Capuchin monks) have been reported in Italy since the 17th century. Different wild edible plants were highly regarded as an important ingredient of this mixed salad. Among these, some species played a key role for both their taste and nutritional properties: *Plantago coronopus* L. (PC), *Rumex acetosa* L., *Cichorium intybus* L., and *Artemisia dracunculus* L. In the present study, the micromorphological and phytochemical features as well as the antioxidant and anti-inflammatory properties of extracts of these fresh and blanched leaves, were investigated. The extracts obtained by blanched leaves, according to the traditionally used cooking method, showed the highest content of bioactive compounds (total phenols 1202.31–10,751.88 mg GAE/100 g DW; flavonoids 2921.38–61,141.83 mg QE/100 g DW; flavanols 17.47–685.52 mg CE/100 g DW; proanthocyanidins 2.83–16.33 mg CyE/100 g DW; total chlorophyll 0.84–1.09 mg/g FW; carbohydrates 0.14–1.92 g/100 g FW) and possess the most marked antioxidant (IC_50_ 0.30–425.20 µg/mL) and anti-inflammatory activity (IC_50_ 240.20–970.02 µg/mL). Considering this, our results indicate that increased consumption of the investigated plants, in particular of PC, raw or cooked briefly, could provide a healthy food source in the modern diet by the recovery and enhancement of ancient ingredients.

## 1. Introduction

Since ancient time, humans have learned to recognize and use wild edible plant species (WEPs) as an important source of supplementary food [1]. Although agricultural production has more than tripled from 1960 to today, also thanks to the technologies of the Green Revolution, widespread hunger and malnutrition still persist in many countries of the world (FAO, 2017).

In this context, the WEPs play a pivotal role in human nutrition, especially in the developing countries, and also represent important components of the Mediterranean Diet today [2,3]. Recent studies have shown a renewed interest in the use of wild plant resources and in the deepening the relationship between plants used as food and their medicinal values [4,5].

Traditional knowledge related to the use of WEPs has been handed down for centuries both orally and in writing in those cultures with a rich literary tradition such as Europe, India, and China [6]. In Europe, from the Middle Ages to the Renaissance, this knowledge was documented, codified, and protected, mainly by monastic communities, within their vegetable gardens (*horti*) [7,8,9,10,11].

The 16th century was a period of progress in European medical knowledge, with renewed interest in healthy foods, such as those vegetables and salads, that until then were considered food of the lower social strata [12].

The Franciscan Order of Capuchin monks (ca 1530) is placed in this cultural context [13,14,15] and Father Zaccaria Boverio in his text *De sacris ritibus* (1626) described an interesting traditional recipe for a mixed salad of wild edible leaves. It was known as “insalatina dei frati” (salad of monks) or “misticanza”, widely used in Liguria and Lazio. According to what was later reported by Father Vincenzo Celesia in *Selva Botanica* (1892), this tradition was still in use at the time among the Ligurian Capuchin monks, who brought this type of healthy salad to their benefactors as a sign of gratitude.

In the present study, we have selected, from this recipe, four species that played a key role for both their taste and nutritional properties, *Plantago coronopus* L. (PC), *Rumex acetosa* L. (RA), *Cichorium intybus* L. (CI), and *Artemisia dracunculus* L. (AD).

*Plantago coronopus* L. (PC), although it is normally considered a weed, has been cultivated for centuries in various traditional contexts for its food and medicinal value, especially from the sixteenth century. Its leaves are cooked as a vegetable in Balkan traditional cuisine, in France and Italy are mixed with other species to prepare salads, with a particular taste and crunchiness. In addition, PC is used in traditional medicine for its analgesic, anti-inflammatory, antipyretic, anticancer and emollient properties, as well as to treat respiratory problems [16,17]. In Liguria PC leaves were in the past the main ingredient of Capuchin monks’ salad, as reported by Father Vincenzo Celesia.

*Rumex acetosa* L. (RA) has been used and cultivated for thousands of years as a medicinal plant, as food and as a dye. Archaeobotanical remains of this plant have been found in Neolithic and Mesolithic settlement sites throughout Europe, where it is still today a very popular vegetable, widely used in traditional cuisine [18,19]. RA is used in folk medicine for its health properties: diaphoretic, diuretic, anti-septic, antipyretic, anti-inflammatory, antioxidant, antimicrobial, anti-hypersensitive, analgesic and antiviral [19,20,21]. In Liguria, still today, the leaves and flowers of RA (“erba agretta”) are chewed against stomatitis [22].

*Cichorium intybus* L. (CI) has been considered a sacred plant and used for its medicinal properties since ancient times [23]. Egyptians, Romans, and Greeks ate chicory root as a useful remedy for liver, digestive, metabolic, and heart ailments. In the Middle Ages, chicory was used to treat jaundice and malaria and its use in European folk medicine has continued until recent times. Chicory root is a traditional remedy for digestive and hepatobiliary diseases, kidney and rheumatic disorders, as well as gout. The aerial parts are mainly used for their diuretic, analgesic, diaphoretic, and antipyretic properties [24,25,26]. In Liguria, leaves of CI are still today consumed as a fresh salad, and briefly boiled as a side dish or to prepare vegetable pies and stuffing of “ravioli”. A decoction of leaves is used for its depurative value [22,27].

*Artemisia dracunculus* L. (AD), native to Siberia and Mongolia, has been introduced in Europe probably since around the XV-XVI centuries, and became a popular spice in culinary tradition for the aromatic taste of its leaves [28,29]. AD has long been used also in traditional medicine as an anti-inflammatory and anti-pyretic, in the treatment of gastrointestinal diseases, and as an anaesthetic, hypnotic and anti-epileptic agent [30,31]. In northern Italy, a still current use of this plant has been reported as a flavouring for making a traditional cheese, called *saurnschotte* [28].

In recent decades, some studies have been conducted on the phytochemical profile of PC, RA, CI, and AD, and their nutritional properties, but much remains to be explored on the antioxidant and anti-inflammatory properties of their extracts, in particular derived from fresh and cooked plants. Nowadays, nutraceutical science shows a growing interest in the recovery and enhancement of ancient ingredients, such as many monastic and conventual wild plants that can be a source of healthy food in the modern diet [32]. Therefore, we carried out the present study, analysing the macro- and micromorphological features and phytochemistry of these species. We also investigated the antioxidant and anti-inflammatory properties of the selected fresh and cooked plant extracts in order to compare their health properties and establish whether the commonly cooking method used affects them, positively or negatively.

## 2. Results and Discussion

### 2.1. Macro- and Micromorphological Characterization 

Recently, the new trend of being green and environmentally friendly has led many people to search for wild edible plants (WEPs), but often this tendency is accompanied by a misidentification between edible and toxic plants, causing poisonings [33,34]. Many WEPs have useful antioxidant and anti-inflammatory properties and are rich in nutritional principles, but their consumption cannot be separated from the correct identification, in order to avoid health risks. Many examples of misidentifications have been reported, such as leaves of *Rumex acetosella* and *R. acetosa* confused with those of *Arum maculatum* or *R. crispus* [35]. Fresh salads are a frequent source of food contamination and poisoning, e.g., *Datura stramonium* accidentally used as ingredient of traditional meals [36], *Digitalis purpurea* and *Mandragora autumnalis* confused with *Borago officinalis* [33,34], and *Veratrum album* mistaken for *Allium ursinum* [37].

Factors contributing to poisoning due to misidentification are often related to the difficulties of identifying the plants in raw mixtures, as well as chopped and processed herbs [38].

**Figure 1:**
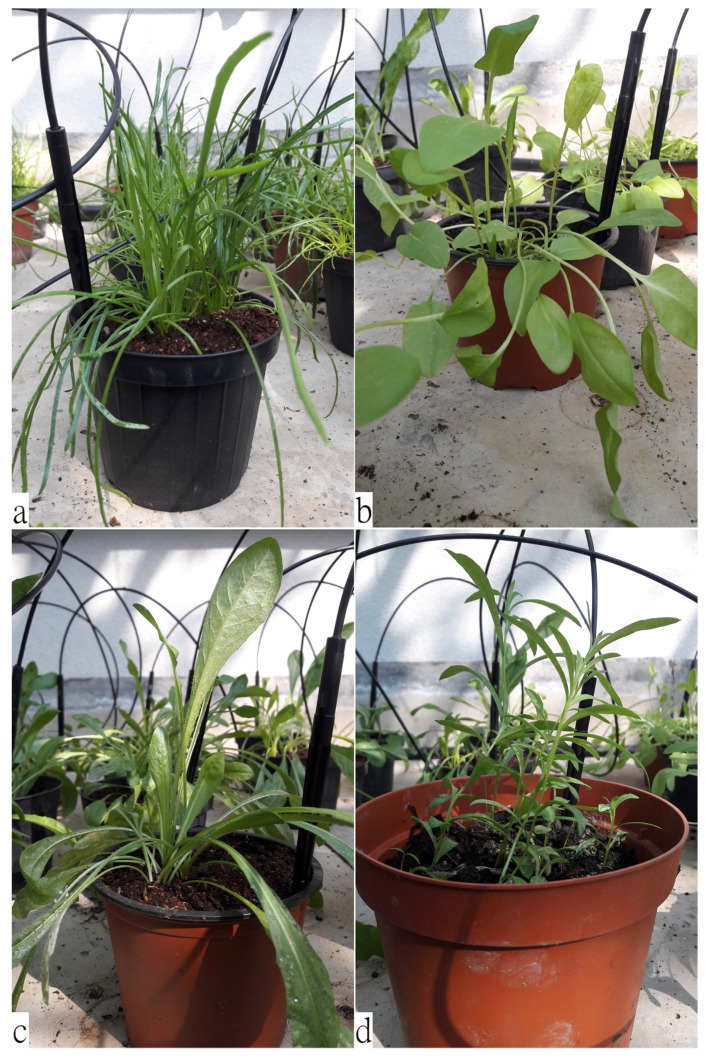
Representative pictures of the four selected plant species: (**a**) *Plantago coronopus* L.; (**b**) *Rumex acetosa* L.; (**c**) *Cichorium intybus* L.; (**d**) *Artemisia dracunculus* L.

**Figure 2:**
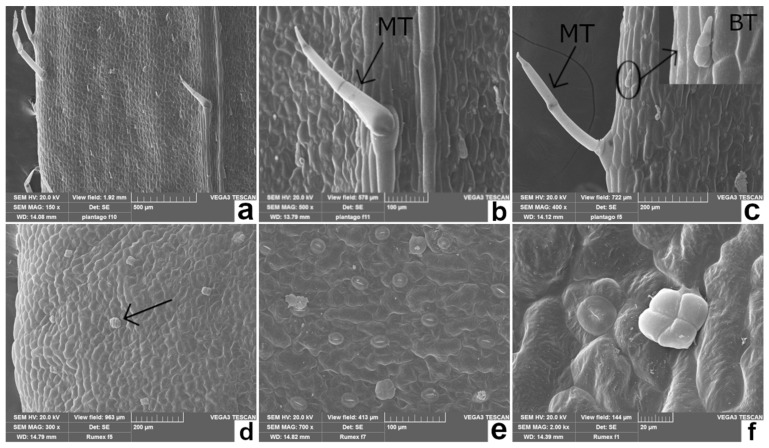
Scanning electron microscopic view of leaves from PC (**a**–**c**) and RA (**d**–**f**). (**a**) Abaxial surface showing epidermal cells, trichomes and stomata; (**b**) abaxial surface showing a non-glandular multicellular trichome (MT) on the leaf midrib; (**c**) adaxial surface showing two types of non-glandular trichomes: bottle-like trichomes (BT) and long stalked MT; (**d**) on the adaxial surface are visible anisocytic and paracytic stomata, and peltate glandular trichomes (the arrow points an abnormal glandular trichome); (**e**) abaxial surface with glandular trichomes and anisocytic and paracytic stomata; (**f**) close-up view of a peltate glandular trichome on the adaxial surface.

**Figure 3:**
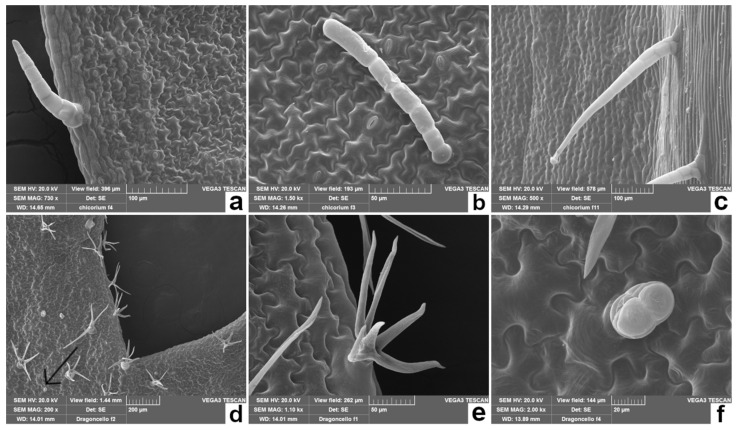
Scanning electron microscopic view of leaves from CI (**a**–**c**) and AD (**d**–**f**). (**a**) Adaxial surface showing a non-glandular multiseriate trichome on the leaf margin; (**b**) adaxial surface showing another type of non-glandular multiseriate trichome and characteristic undulating epidermal cell walls; (**c**) abaxial surface showing multiseriate glandular trichomes along the midrib; (**d**) abaxial surface showing stellate non-glandular trichomes and biseriate glandular trichomes (arrow); (**e**) close-up view of a stellate non-glandular trichome; (**f**) close-up view of a biseriate glandular trichome.

The edible leaves of the species considered in our study have a significant intraspecific morphological variability as a plastic response to different environmental conditions [19,29,39,40]. Therefore, macromorphological analysis combined with scanning electron microscopy (SEM) (Table 1 and Figure 1, Figure 2 and Figure 3) allowed one to highlight some anatomical details of PC, RA, CI, and AD, that can be used for the taxonomic determination of the plant material, even when the leaves are mixed in salad, frozen, or processed. In particular, different typical features of the epidermal surfaces of each species were pointed out, including the shape of the epidermal cells, the type of trichomes, and the stomatal apparatus.

### 2.2. Phytchemical Investigations

The edible leaves of the selected plants, once characterized from the micromorphological point of view, were processed, partly fresh and partly blanched, by pulverization with liquid nitrogen and extraction by sonication. These treatments allowed one to obtain high extractive yields, between 1.40% and 4.10%, without modifying the native phytochemical profile of the investigated plants, by exhaustively extracting the bioactive compounds. The phytochemical screening carried out made it possible to quantify the total phenols, flavonoids, flavanols (vanillic index) and proanthocyanidins present in the four plant species of interest, also allowing to calculate the polymerization index, i.e., the preponderance of monomeric/polymeric molecules in each extract. Moreover, since it is well known that pigments, especially chlorophyll and carbohydrates, can contribute to the biological properties of these matrices and may, more or less, be affected by the cooking method used [47], even the latter have been determined on the starting matrices, as they are and after blanching 

As reported in Table 2, all extracts analyzed showed a very interesting phytochemical profile, being very rich in bioactive compounds. 

Specifically, the results obtained with the fresh extracts and those obtained with the blanched extracts were compared with each other, and then the results obtained with each fresh extract was compared to the corresponding blanched extract.

Already in the extracts obtained from fresh leaves, important differences were found. PCF, in particular, showed the highest content of total phenols, flavonoids, flavanols and carbohydrates with statistically significant results (*p* < 0.05) compared to all other plant species investigated (Table 2). The total phenols content, in particular, is about double with respect to that previously reported by Janković et al. [48], who observed a total phenols content for *P. coronopus* equal to 925 mg GAE/100 g DE.

The other plants, on the contrary, showed a very variable trend depending on the class of bioactive compound investigated. With respect to the total phenols content, ADF resulted as the richest one, followed by CIF and RAF, while it was the poorest in terms of flavonoids after RAF and CIF. However, examining the class of flavanols, it appears again as the richest one after PCF, RAF and CIF. Finally, in terms of proatocyanidins, RAF was found to be the richest extract, followed by CIF, PCF and ADF.

On the contrary, CIF and ADF represent the richest source of chlorophyll without any statistically significant difference between them, followed by RAF and PCF (*p* < 0.05). Moreover, CIF contains the highest content of carbohydrates after PCF (*p* < 0.05), followed by ADF and RAF (*p* < 0.05) (Table 2).

Surprisingly, the leaf blanching statistically, and significantly, increased (*p* < 0.05) the bioactive compounds content in all the extracts investigated, with the exception of the total phenols, chlorophyll and carbohydrates content in ADF, which showed comparable amounts. The same trend was observed also for PCF, but only for chlorophyll content (Table 2).

Indeed, apart from the total chlorophyll and carbohydrate content of RAC and CIC, whose decreased in a statistically significant manner after blanching with respect to RAF and CIF (*p* < 0.05), and ADC, which remained unchanged, in all other cases, the blanched leaf extracts showed a bioactive compounds content from 1.2 to 13 times higher than the fresh leaf extracts (Table 2). 

These results are in accordance with previous studies, which highlighted that the blanching process resulted in a significant increase in total phenols and flavonoids as result of their easily extraction by plant cell membrane disruption [49,50,51,52]. Similar observations, in particular, were reported also for coriander and majorana leaves [51,53]. During blanching, indeed, exposure to high-temperature steam can cause tissue disruption and the release of polyphenols from the vacuole or other cellular structures. However, this phenomenon, and consequently the extent of tissue damage, is strictly related to the plant’s heat tolerance [54]. Furthermore, the degree to which phytochemicals change during processing depends on their structure and, consequently, by the sensitivity of the compound to the heat-induced modification or degradation [55]. The only decrease was recorded in terms of the proanthocyanidins content in RAC. It must be said, however, that RAF, the corresponding fresh leaf extract, showed the highest proanthocyanidins content, compounds well known to give depolymerization after heating leading to an increase in terms of monomeric polyphenols [56].

This phenomenon appears even more evident calculating the polymerization index (vanillin index/proanthocyanidins). Indeed, the extracts obtained from fresh leaves have a significantly higher polymerization index (*p* < 0.05) than the corresponding extracts obtained from blanched leaves (350.62 vs. 34.13 for PCF and PCC, respectively; 8.96 vs. 4.62 for RAF and RAC, respectively; 2.89 vs. 1.83 for CIF and CIC, respectively; 2028.95 vs. 242.15 for ADF and ADC, respectively) having a much higher flavanols content than proanthocyanidins. However, after blanching, this index is significantly reduced as a result of a substantial increase in the proanthocyanidins content and a more restrained flavanol content, leading to a greater presence of monomeric molecules. These results are in accordance with White et al. [50], who observed a significant increase in both proanthocyanidins oligomers and polymers after blanching.

From the calculation of the polymerization index, it is possible to deduce also that AD is the plant characterized by the greatest number of polymeric molecules, followed by PC, RA and CI.

The plant species investigated showed a comparable amount of carbohydrates with respect to previous studies carried out on fresh salads [57], and a higher content of total chlorophyll with respect to previous investigations (1.04 and 0.93 mg/g vs. 0.56 and 0.76 mg/g for RA and PC, respectively; 1.14 mg/g vs. 0.30 mg/g for CI; 1.13 mg/g vs. 0.89 mg/g for AD) [58,59,60] that could be attributed to the freshness and young leaves used in the present study, as well as also by the speed with which they were processed after collection. 

Even in the case of chlorophyll and carbohydrates, the results reflect those reported in the literature, which state that rapid blanching does not influence or has very little influence on the content of these substances, with a sometimes-significant increase in the same after cooking, which allows an increase in the biological activity observed [61,62].

To conclude, after blanching, all plant leaves investigated, showed a higher content of bioactive compounds, albeit with substantial differences in some cases in terms of the phytochemical profile. However, among the investigated species, PC continues to be the extract that shows the highest content of total phenols, flavonoids, flavanols, chlorophyll and carbohydrates, certainly proving to be the most interesting plant in terms of phytochemical profile, becoming also the richest one in proanthocyanidins following cooking.

### 2.3. Health Properties

The extracts obtained by fresh and blanched plant leaves were also characterized from a biological point of view using a battery of antioxidant and anti-inflammatory tests based on different environments and reaction mechanisms. This is very important, since, given the complexity of a plant extract in terms of phytochemicals, it is impossible to evaluate its antioxidant and anti-inflammatory activity with a single method [52]. Considering this, four different antioxidant assays based on electron transfer (FRAP), electrons’ and hydrogen atoms’ transfer (TEAC), hydrogen atom transfer (ORAC) and iron-chelating activity, were carried out. Regarding anti-inflammatory activity, the heat-induced BSA denaturation assay (BDA) and the protease inhibitory activity test (APA), were carried out. The first one is based on the sample’s ability to protect endogenous proteins against denaturation, whereas the second one is based on the sample’s ability to inhibit directly the protease, an enzyme well-known to be involved in several inflammatory-based diseases [63].

The results of the health properties of the extracts under examination, expressed as IC_50_ (µg/mL) with the respective C.L., are shown in Table 3. Additionally, in this case, the results obtained for the fresh extracts, as well as those obtained from the blanched extracts were compared between them. Furthermore, the results of each fresh extract were compared with those obtained with the extract of the respective blanched leaves.

**Figure 4:**
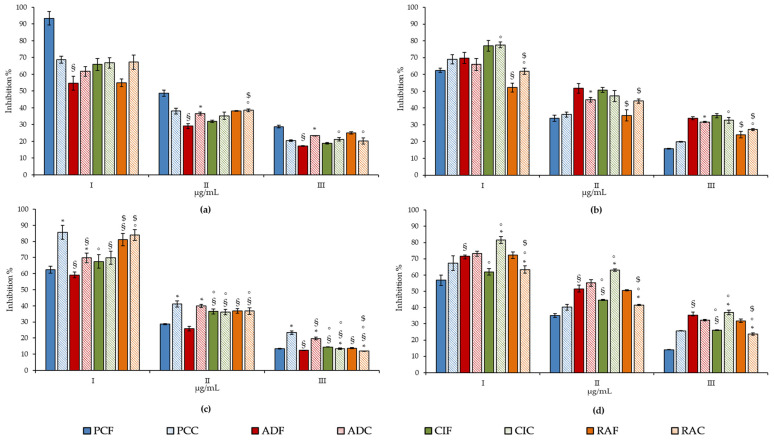
Antioxidant and free radical-scavenging activity of fresh (F) and cooked (C) leaf extracts of the traditional wild salad of the Capuchin monks: *Plantago coronopus* L. (PC), *Rumex acetosa* L. (RC), *Cichorium intybus* L. (CI), and *Artemisia dracunculus* L. (AD). Results were expressed as mean inhibition percentage (%) ± standard deviation of three independent experiments (*n* = 3). (**a**) FRAP, concentration ranges (I–III): 12.5–50.0 μg/mL for PCC; 150.0–600.0 μg/mL for PCF, RAC, CIC, ADF and ADC; 300.0–1200.0 μg/mL for CIF; 600–2400.0 μg/mL for RAF; (**b**) TEAC, concentration ranges (I-III): 12.5–50.0 μg/mL for PCC; 80.0–320.0 μg/mL for PCF; 150.0–600.0 μg/mL for ADC, ADF, CIC and RAC; 300.0–1200.0 μg/mL CIF and RAF; (**c**) Ferrozine, concentration ranges (I–III): 3.0–12.0 μg/mL for PCF, RAF, RAC, CIF, CIC, ADF and ADC; 12.0–48.0 μg/mL for PCC; (**d**) ORAC, concentration ranges (I–III): 0.12–0.50 μg/mL for PCC; 0.5–4.0 μg/mL for PCF, CIF, CIC, ADF, ADC and RAC; 1.5–12.0 μg/mL for RAF. * *p* < 0.05 vs. fresh extract; ^§^ *p* < 0.05 vs. PCF or PCC; ° *p* < 0.05 vs. ADF or ADC; ^$^ *p* < 0.05 vs. CIF or CIC.

**Figure 5:**
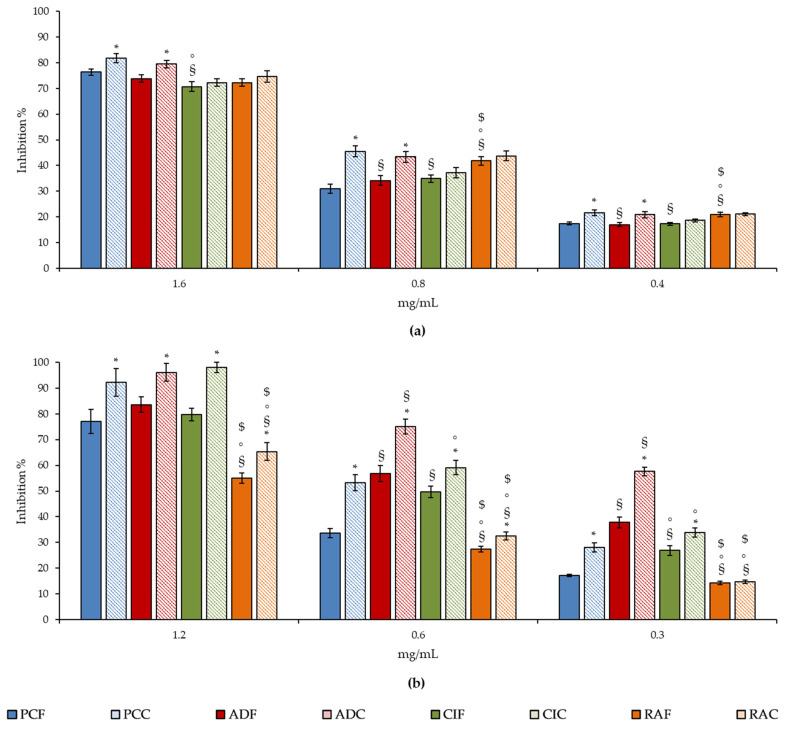
Anti-inflammatory activity of fresh (F) and cooked (C) leaf extracts of the traditional wild salad of the Capuchin monks: *Plantago coronopus* L. (PC), *Rumex acetosa* L. (RC), *Cichorium intybus L*. (CI), and *Artemisia dracunculus* L. (AD) towards BSA denaturation assay (**a**) and protease inhibition assay (**b**). Results were expressed as mean inhibition percentage (%) ± standard deviation of three independent experiments (*n* = 3). * *p* < 0.05 vs. fresh extract; ^§^ *p* < 0.05 vs. PCF or PCC; ° *p* < 0.05 vs. ADF or ADC; ^$^ *p* < 0.05 vs. CIF or CIC.

All the samples showed a marked and concentration-dependent antioxidant and anti-inflammatory activity (Figure 4 and Figure 5, respectively), although there are important and statistically significant differences between the different extracts investigated, both as fresh and blanched extracts (Table 3). 

First of all, when comparing the results of the extracts obtained from fresh leaves, the same order of activity appears evident, with PCF, which showed the strongest antioxidant activity, followed by ADF, CIF and RAF, both in the TEAC and FRAP test (Table 3 and Figure 4). The situation changes radically instead in the ORAC test, where ADF shows the highest antioxidant activity, followed by CIF, PCF and RAF, which holds the last position (Table 3 and Figure 4). On the contrary, RAF exhibits the strongest iron-chelating activity, followed by CIF, ADF and PCF (Table 3 and Figure 4). This order of potency positively correlates with the total phenols and flavonoids content, and the slight differences found in terms of activity between the extracts under study can be attributed to the greater or lesser expression of some secondary metabolites, as seen in Table 2, as well as from the specific chemical structures of the polyphenols present [51].

What is certain, however, is that the marked iron-chelating activity found in RAF is attributable to the high proanthocyanidins content found in this extract. Indeed, it has been shown, both in vitro and in vivo, that proanthocyanidins, thanks to their strong iron-chelating activity, act as antimicrobial agents [64] and protect against oxidative renal damage induced by iron overload in rats [65].

Despite that no statistically significant difference was found between the extracts under examination, in terms of the inhibition of heat-induced protein denaturation (BDA) (Table 3), also in this case, the proanthocyanidins content seems to play a pivotal role in the activity of RAF extract, which shows the lowest IC_50_ value and, therefore, the most marked anti-inflammatory activity, followed by PCF, CIF and ADF. The activity found, attributable to an anti-peroxidase activity of the extract under study, has been previously observed for other proanthocyanidins-rich extracts [66].

On the contrary, the strong anti-tryptic activity was recorded for ADF, followed by CIF, PCF and RAF. However, specifically, a statistically significant difference was found only between RAF, CIF and ADF (Table 3 and Figure 5). This activity seems to correlate mainly with the total content of phenols and flavanols, although what makes the difference is the structure of the main polyphenols present in the extracts under examination, rather than the class of compounds. Indeed, being an enzymatic activity, it implies a direct interaction of the bioactive compound/s at the enzyme level.

Finally, it is interesting to note that, in accordance with what has been observed for the phytochemical characterization, the extracts obtained from blanched leaves show a much more pronounced free-radical scavenging (TEAC, FRAP and ORAC assay) than the extracts obtained from fresh leaves, with statistically significant results for all tested extracts except ADC (*p* < 0.05). On the contrary, no statistically significant difference was found as regards the iron-chelating activity and the anti-inflammatory activity, in which the extracts obtained from the cooked leaves did not show a statistically significant difference compared to the corresponding extracts obtained from the fresh leaves.

The increase or retention of total phenols, flavonoids and proanthocyanidins content, as well as of the antioxidant and anti-inflammatory activity of plant extracts during blanching, may be mainly ascribed to the increase of individual polyphenols or to their thermal degradation through, for example, deglycosylation or hydrolysis processes, which contribute differently, with respect to the parent compounds, to the health properties investigated [50]. Although an in-depth phytochemical characterization is outside the topic of this manuscript, comparing completely different plant species from a phytochemical point of view, recent studies have shown that the plants subject to this study are particularly rich in complex polyphenols, mostly glycosylated and esterified. In particular, *P. coronopus* is characterized by the iridoid glucosides, aucubin and catalpol, as well as the phenylpropanoid glycoside, acteoside. Among flavonoids, luteolin- and apigenin-7-*O*-glucoside represent the most abundant compounds [48]. The phytochemical profile of *R. acetosa* includes anthraquinones, polyphenols and a high level of oxalic acid, which seems to be reduced to a negligible amount during cooking. The main phenolic compounds present include resveratrol, vanillic and sinapic acid and catechin. The leaves contain also β-carotene, but not in a significant amount for human health [18]. The phytochemical profile of *A. dracunculus* showed, apart from the characteristic lactones, artemisinin, dihydroartemisinin and artemether, several flavonoids among which the most abundant are rutin, luteolin, naringenin and chrysin [67]. Regarding *C. intybus*, other than some simple phenolics such as malic, caffeic, quinic, caftaric and chlorogenic acid, all identified components were in the glycosylated or ester form. Among these, the most abundant are the glycosylated derivatives of cyanidin, delphinidin, quercetin, kaempferol, isorhamnetin and apigenin. Furthermore, acetyl and malonyl derivatives were also found [68,69].

In addition, the contribution of non-phenolic substances such as sugars or ascorbic acid must not be neglected, because they can contribute in particular to the reducing ability of the plant extracts. Finally, synergistic and additive effects of polyphenols may enhance the biological activity observed [51].

## 3. Materials and Methods

### 3.1. Plant Material and Growth Conditions

Seeds of PC, RA, CI, and AD were purchased from Fratelli Ingegnoli Spa (Milano, Italy) and sown according to Poorter et al. [70].

**Figure 6:**
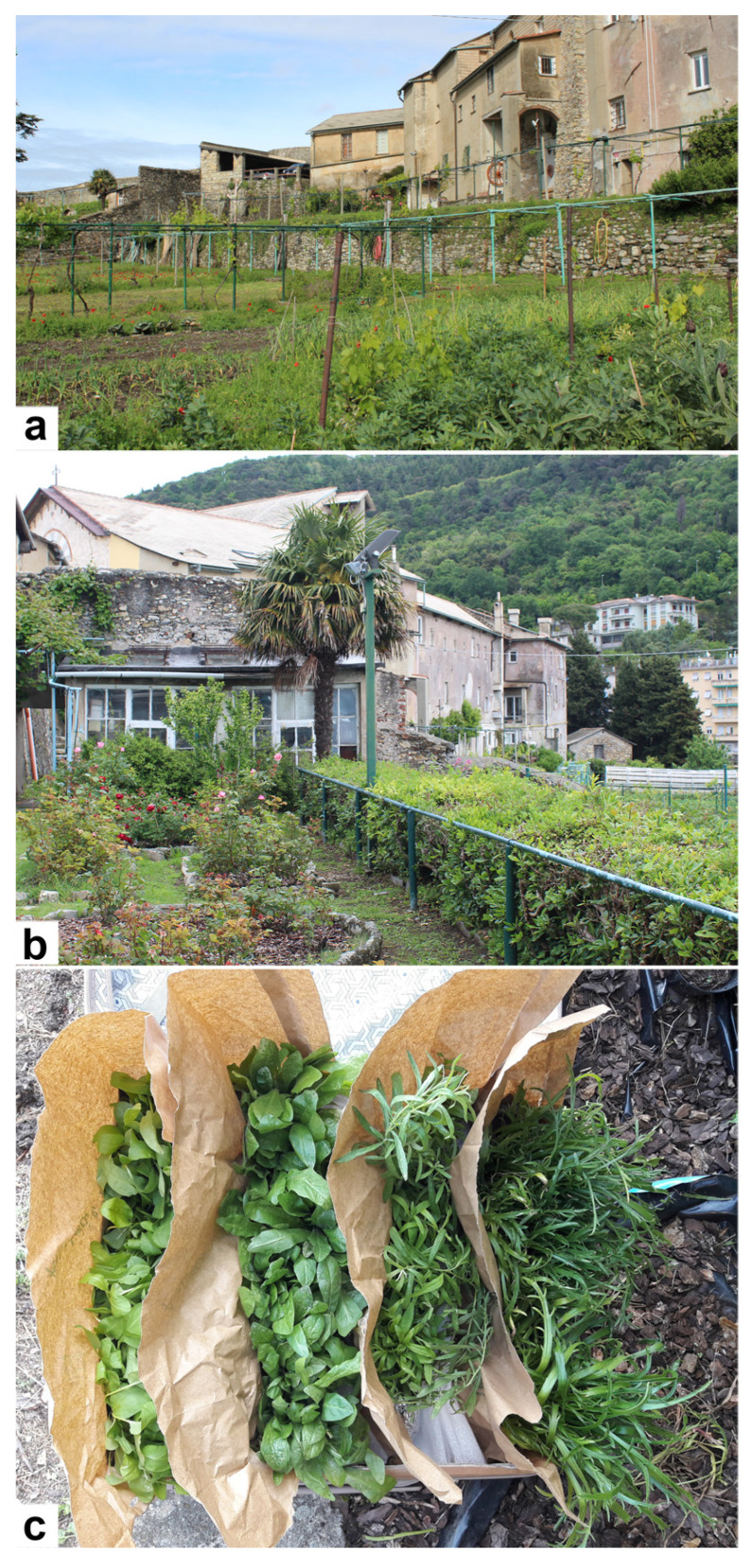
(**a**,**b**) San Barnaba Convent vegetable garden and greenhouses (Genova, Italy); (**c**) plants collected after 60 days for carrying out laboratory analyses.

Briefly, they were sown in plastic plug within a greenhouse located in San Barnaba convent (Genova, Italy, 190masl; 44°25′26″ N 8°55′42″ E, https://goo.gl/maps/UaN9eCS9cWtQvcT56, accessed on 22 January 2022) (Figure 6a,b) and equipped with additional UV artificial lighting to ensure constant daily irradiation and fine-textured shade-nets to attenuate the excessive solar overheating according to Lenka et al. [71].

Plastic plug trays were filled with Irish peat (Vigorplant Italia Srl) and after fifteen days 1 g/L NPK 20-10-20 water soluble fertilizer (Vialca Srl) was added once a week. In the first two weeks, plants were watered (about 180 mL) twice a day for three days a week (Monday, Wednesday, Friday). Subsequently, they were watered once a day, only on Monday and Friday.

After two months, the plant leaves were collected (Figure 6c) and immediately sent to the laboratory, where they were suitably processed for micromorphological, phytochemical and biological analyses.

### 3.2. Scanning Electron Microscopy (SEM) Analysis

Plant leaves were cut, using a razor blade, into small pieces (15–20 mm^2^). Such dimensions have allowed to have sufficient surfaces of both leaf pages to analyze the distinctive characters of the selected species. Samples were fixed in FineFIX working solution (Milestone s.r.L., Bergamo, Italy) with 70% ethanol, and left overnight at 4 °C [72]. The next day, samples were serially dehydrated in ethanol (80, 90, 95 and 100%) for 1 h, and then in CO_2_ using a Critical Point Drier processor (K850 CPD 2M Strumenti S.r.l., Roma, Italy).

Dried specimens were mounted on stubs using double stick tape and coated with 10 nm gold. SEM analysis was carried out using a Vega3 Tescan LMU (Tescan USA Inc., Cranberry Twp, PA, USA) at an accelerating voltage of 20 kV.

### 3.3. Mixed Salad Traditional Recipe

The plants analyzed in this study are part of the recipe of a mixed salad reported by Zaccaria Boverio in 1626 [73]. These plants were also cited in other contemporary texts dealing with salads, such as *Archidipno* by Salvatore Massonio (1627) [74]. According to Zaccaria Boverio, the plants used in the Capuchin monks mixed salad were: Coronopum (*Plantago coronopus* L.), Intubum (*Cichorium intybus* L.), Oxalim (*Rumex acetosa* L.), Draconculum (*Artemisia dracunculus* L.), Pimpinellam (*Poterium sanguisorba* L.), Lactucas crispas (*Lactuca sativa* L. var. crispa), Lactucas laconicas (*Lactuca sativa* L. var. capitata), Nasturcium (Nasturtium officinale W.T. Aiton), Rapa sylvestria (*Brassica rapa* L. subsp. sylvestris (L.) Janch.) and Mentam (*Mentha* spp.). This mixed salad was known as “insalatina dei frati”.

One of the most frequently reported cooking methods throughout history has been represented by a short boiling or blanching. This method is still commonly mentioned in ethnobotanical studies concerning the use of WEPs in Mediterranean area [75]. In addition, it is used both at domestic level and in the food industry to inactivate enzyme activity and to preserve vegetables [76].

### 3.4. Chemicals

All reagents and solvents were of analytical grade and, as well as trolox, were purchased from Merck (Darmstadt, Germany). Standard compounds (gallic acid, quercetin, catechin, cyanidin chloride) were purchased from Extrasynthese (Genay, France).

### 3.5. Sample Processing and Extract Preparation

For each plant species collected (PC, RA, CI, and AD), leaves were cut, gently cleaned with paper and weighed (50 g). In order to obtain the fresh extracts (PCF, RAF, CIF, and ADF), the leaves were added directly in a blade mill pulverization chamber (A11, IKA^®^-Werke GmbH & Co. KG, Staufen, Germany) to which was added liquid nitrogen during powdering, in order to preserve the nutritional and chemical features by blocking enzymatic activities. For cooked plant extracts (PCC, RAC, CIC, and ADC), leaves, before powdering according to the above protocol, were added to boiling water and blanched for 1 min in compliance with the most traditionally used cooking method.

Thereafter, powdered raw or cooked leaves (10 g for each plant species) were added with 100 mL 70% ethanol, mixed for 3 min and then sonicated in ice-cold bath for 5 min using a 3 mm titanium probe set to 200 W and 30% amplitude (Vibra Cell™ Sonics Materials, Inc., Danbury, CT, USA). Samples were centrifuged at 3000× *g* for 15 min at 4 °C and the supernatants were evaporated to dryness by a rotary evaporator at RT. The extraction procedure was repeated 3 times. Dry extracts (DE) were stored for 24 h in a vacuum desiccator in the dark on anhydrous sodium sulphate and subsequently stored at −20 °C until the subsequent analyses, which were carried out solubilizing and appropriately diluting the dry extracts in the extraction solvent (70% ethanol).

### 3.6. Phytochemical Screening

#### 3.6.1. Total Phenols

Total phenols were quantified according to Bazzicalupo et al. [77] using the Folin–Ciocalteu reagent, by mixing each extract solution (6.0–24.0 mg/mL for PCF, RAF, RAC, CIF, CIC, ADF and ADC; 0.5–2.0 mg/mL for PCC) with deionized water and Folin–Ciocalteu reagent in the following ratio: 1:9:10, *v*/*v*/*v*. After 3 min, 10% sodium carbonate (1:2, *v*/*v*) was added and samples were incubated for 60 min in the dark at RT, mixing every 10 min. The absorbance was recorded at 785 nm with a UV-VIS spectrophotometer (Shimadzu UV-1601, Kyoto, Japan). Gallic acid was used as reference compound (0.075–0.60 mg/mL) and results, which represent the average of three independent experiments in triplicate (*n* = 3), were expressed as mg of gallic acid equivalents (GAE)/100 g DE.

#### 3.6.2. Flavonoids

The flavonoid content was quantified according to Smeriglio et al. [78]. Briefly, 200 µL of each extract solution (3–12.0 mg/mL for PCF, RAF, CIF, CIC and ADF; 6.0–24.0 mg/mL for ADC and RAC; 0.20–0.80 mg/mL for PCC) were added to 2 mg/mL AlCl_3_ (1:1, *v*/*v*), and brought up to 1.6 mL with 50 mg/mL sodium acetate. After 2.5 h, the absorbance was recorded at 440 nm using an UV–Vis spectrophotometer (Shimadzu UV-1601, Kyoto, Japan). Quercetin was used as reference compound (0.25–1.0 mg/mL) and results, which represent the average of three independent experiments in triplicate (*n* = 3), were expressed as mg of quercetin equivalents (QE)/100 g DE.

#### 3.6.3. Vanillin Index

The flavanols content was evaluated according to Boudjelal et al. [79] by vanillin index test. Briefly, 2.0 mL of sample solution, diluted in 0.5 M H_2_SO_4_ in order to reach an absorbance ranging from 0.2 to 0.4 (1.75 mg/mL for ADC; 3.5 mg/mL for PCF, PCC and ADF; 6.25 mg/mL for RAC, CIF and CIC; 12.5 mg/mL for RAF), were loaded onto a conditioned Sep-Pak C18 cartridge (Waters, Milan, Italy). The column was activated by 2.0 mL of 5.0 mM H_2_SO_4_ and then air-purged. Samples were slowly eluted by adding 5.0 mL of methanol. Six millilitres of a 4% vanillin methanol solution were added to 1 mL of sample eluate, incubating in a water bath at 20 °C for 10 min. After this, 3 mL of HCl was added and after 15 min, the absorbance was recorded at 500 nm with a UV-VIS spectrophotometer (Model UV-1601, Shimadzu, Kyoto, Japan) against a blank consisting of the same sample solvent. Catechin was used as reference compound (0.125–0.50 mg/mL). Results, which represent the average of three independent experiments in triplicate (*n* = 3), were expressed as g of cathechin equivalents (CE)/100 g DE.

#### 3.6.4. Proanthocyanidins

The proanthocyanidin content was evaluated according to Baali et al. [80]. Briefly, 2.0 mL of sample solution diluted with 0.05 M H_2_SO_4_ (0.75 mg/mL for PCC; 5 mg/mL for PCF, PCC, ADF and ADC; 10 mg/mL for RAC and CIF; 20 mg/mL for RAF), was loaded onto a Sep-Pak C18 cartridge (Waters, Milan, Italy) preconditioned with 5 mM H_2_SO_4_ (2.0 mL), and purged with air. Samples were eluted with methanol (3.0 mL) and collected in a 100 mL flask shielded from light, containing 9.5 mL of absolute ethanol. After that, 12.5 mL of FeSO_4_ · 7H_2_O solubilized in 37% HCl (300 mg/L) was added to the reaction mixture and placed to reflux for 50 min. After cooling by immersion in cold water (20 °C) for ten min, the absorbance was read at 550 nm with an UV-VIS spectrophotometer (Model UV-1601, Shimadzu, Kyoto, Japan) against a blank consisting of the same sample solvent. The basal anthocyanins content of samples was determined detracting the absorbance of samples prepared under the same conditions reported above, without the reflux process. Proanthocyanidins content was expressed as 5 times the amount of cyanidin formed by means of a cyanidin chloride (ε = 34,700) calibration curve. Results, which represent the average of three independent experiments in triplicate (*n* = 3), were expressed as mg of cyanidin equivalents (CyE)/100 g DE.

#### 3.6.5. Chlorophyll Determination

The total chlorophyll content (chlorophyll a + chlorophyll b) was evaluated according to Porra et al. [81]. Briefly, 0.2 g of leaves of fresh and blanched PC, RA, CI and AD were homogenized in an ice-cold mortar with quartz sand by adding 1 mL of 80% acetone for three times. Samples were then poured in a screw cap glass tube, washing with 1 mL of acetone, and centrifuged at 3000× *g* for 10 min, 4 °C. The supernatants were recovered with a glass Pasteur pipette and stored in a graduated glass cylinder in the dark. The sample pellets were resuspended with 2 mL of 80% acetone and centrifuged as described above. This procedure was repeated until exhaustive extraction. Finally, the supernatants were pooled in the cylinder and the volume noted to calculate the dilution factor. The absorbance was recorded at 663 nm to quantify chlorophyll *a*, and at 648 nm to quantify chlorophyll *b* content, according to the following equations:

Chlorophyll a (µg/mL)=12.25×ABS(663 nm)−2.55×ABS (648 nm)

Chlorophyll b (µg/mL)=20.31×ABS(648 nm)−4.91×ABS (663 nm)

Results, which represent the average of three independent experiments in triplicate (*n* = 3), were expressed as mg/g of fresh weight (FW).

#### 3.6.6. Carbohydrates

The carbohydrates content was determined according to Bazzicalupo et al. [77]. Briefly, 10 mg of fresh and blanched PC, RA, CI and AD were placed in a glass test tube with screw cap, together with 0.5 mL of 2.5 mol/L HCl, and the mixture was incubated in a water bath at 100 °C for 3 h. After cooling, samples were neutralized with sodium carbonate and brought up to 10 mL with distilled water. After centrifugation at 3500× *g* for 5 min, 20 µL of each sample, blank (distilled water) or reference standard (glucose 5–100 µg/mL), were brought up to 200 µL with distilled water. Two-hundred microliters of 5% phenolic solution and 1 mL of 96% H_2_SO_4_ were added and the reaction mixture was stirred for 10 min and incubated in a water bath at 30 °C for 20 min. The absorbance was recorded at 490 nm and results, which represent the average of three independent experiments in triplicate (*n* = 3), were expressed as g/100 g of fresh weight (FW).

### 3.7. Evaluation of Health Properties: Antioxidant and Anti-Inflammatory Activity

The health properties of fresh and cooked extracts were evaluated by several colorimetric in vitro cell-free assays based on different reaction mechanisms and environments. Absorbance and fluorescence data, acquired by a UV-VIS (Multiskan GO; Thermo Scientific, MA, USA) and a fluorescence plate reader (Fluostar Omega, BMG labtech, Ortenberg, Germany), respectively, were recorded against a blank consisting of samples’ extraction solvent (70% ethanol). Results, which represent the average of three independent experiments in triplicate (*n* = 3), were expressed as half-maximal inhibitory concentration (IC_50_, μg/mL) with confident limits (CLs.) at 95%, calculated by the Litchfield and Wilcoxon test, using PHARM/PCS software version 4 (MCS Consulting, Wynnewood, PA, USA). Sample and reference compound concentration ranges reported below refer to the final concentrations into the reaction mixture, which did not show any interference at the characteristic wavelengths of the tests carried out.

#### 3.7.1. Antioxidant Activity

##### Trolox Equivalent Antioxidant Capacity (TEAC) Assay

The TEAC test was performed according to the method described by Smeriglio et al. [82], with some modifications. Briefly, the reagent solution consisting of 1.7 mM ABTS and 4.3 mM (NH_4_)_2_S_2_O_8_ was incubated in the dark at RT for 12 h, diluted with deionized water until an absorbance of 0.7 ± 0.02 at 734 nm, and used within 4 h. Five microliters (5 μL) of each sample solution (12.5–50.0 μg/mL for PCC; 80.0–320.0 μg/mL for PCF; 150.0–600.0 μg/mL for ADC, ADF, CIC and RAC; 300.0–1200.0 μg/mL CIF and RAF) were added to 100 µL of the reagent solution, mixed and incubated in the dark for 6 min at RT. The absorbance was recorded at 734 nm using trolox (1.0–4.0 μg/mL) as reference compound.

##### Ferric Reducing Antioxidant Power (FRAP) Assay

The FRAP test was carried out according to Muscarà et al. [83], with some modifications by using a fresh pre-warmed (37 °C) reagent consisting of 300 mM buffer acetate (pH 3.6), 10 mM 2,4,6-Tris (2-pyridyl)-s-triazine (TPTZ)-40 mM HCl, and 20 mM FeCl_3_. Briefly, 5 μL of each sample solution (12.5–50.0 μg/mL for PCC; 150.0–600.0 μg/mL for PCF, RAC, CIC, ADF and ADC; 300.0–1200.0 μg/mL for CIF; 600.0–2400.0 μg/mL for RAF) were incubated for 4 min with 100 µL of the above reagent and the absorbance was recorded at 593 nm by using trolox (1.0–4.0 μg/mL) as reference compound.

##### Oxygen Radical Absorbance Capacity (ORAC) Assay

The ORAC test was carried out according to Smeriglio et al. [84]. Twenty microliters of sample solution (0.12–0.5 μg/mL for PCC; 1.0–4.0 μg/mL for PCF, CIF, CIC, ADF, ADC and RAC; 3.0–12.0 μg/mL for RAF) diluted in 75 mM phosphate buffer (pH 7.4), were mixed with 117 nM fresh fluorescein solution (120 μL) and incubated for 15 min at 37 °C, before adding 40 mM fresh AAPH solution (60 μL) to start the reaction. The probe decay was monitored for 90 min by recording the fuorescence intensity every 30 s using the following excitation and emission wavelengths: λ_ex_ 485 nm and λ_em_ 520 nm. Trolox (0.25–1.0 μg/mL) was used as reference compound.

##### Iron-Chelating Activity

Iron-chelating activity was evaluated by ferrozine assay according to Muscarà et al. [85], with some modifications. Five microliters of each sample solution (3.0–2.0 μg/mL for PCF, RAF, RAC, CIF, CIC, ADF and ADC; 12.0–48.0 μg/mL for PCC), ethylenediaminetetraacetic acid (EDTA) as reference standard (3.0–12.0 μg/mL), or blank (70% ethanol), were added to 2.5 μL of 2 mM FeCl_2_ · 4 H_2_O and incubated at RT for 5 min. After that, 5 μL of 5 mM ferrozine and 137.5 μL of deionized water were added to the reaction mixture. The absorbance was recorded after 10 min at 562 nm.

#### 3.7.2. Anti-Inflammatory Activity

##### Bovine Serum Albumin (BSA) Denaturation Assay

The BSA denaturation assay was carried out according to Denaro et al. [86]. Briefly, 80 μL of each sample solution (0.075–0.60 mg/mL for CIF and CIC; 0.15–1.20 mg/mL for ADF, ADC, RAC, PCF and PCC; 1.2–9.60 mg/mL for RAF) were added to 100 μL of 0.4% BSA fatty acid-free solution and 20 μL of phosphate-buffered saline (PBS, pH 5.3). The absorbance was recorded at 595 nm at the starting time (T_0_) and after incubation for 30 min at 70 °C in order to measure the samples’ ability to counteract the heat-induced BSA denaturation. Diclofenac sodium (15.63–62.50 μg/mL) was used as a reference compound.

##### Protease Inhibition Assay

The anti-tryptic activity was evaluated according to Smeriglio et al. [87]. Briefly, 200 μL of each sample solution (0.20–1.60 mg/mL for PCF, PCC, ADF, ADC, CIF, CIC, RAF and RAC) was added to the reaction mixture consisting of 12 μL trypsin (10 μg/mL) and 188 μL Tris-HCl buffer (25 mM, pH 7.5). After that, 200 μL of 0.8% casein was added to the reaction mixture, starting the incubation time (20 min, 37 °C). Reaction was stopped by adding 400 μL perchloric acid, which allowed the protein precipitation, leading to a cloudy suspension, which was centrifuged at 3500× *g* for 10 min. The absorbance of the supernatant was recorded at 280 nm. Diclofenac sodium (15.63–62.50 μg/mL) was used as reference compound.

### 3.8. Statistical Analysis

Results, which represent the average of three independent experiments in triplicate (*n* = 3), were expressed as mean ± standard deviation (S.D.) for phytochemical analyses, and as IC_50_ with CLs. at 95% for health properties evaluation. Data were analysed by one-way analysis of variance (ANOVA) followed by Dunnett’s test for antioxidant and anti-inflammatory assays, and Tukey’s test for phytochemical screening by SigmaPlot 12.0 software (Systat Software Inc., San Jose, CA, USA). Results were considered statistically significant for *p* < 0.05.

## 4. Conclusions

In conclusion, the investigated ingredients of the Capuchin monks’ mixed salad represent a good source of polyphenols with interesting antioxidant and anti-inflammatory properties if consumed both raw or briefly cooked. Blanching affects the bioactive compounds content, both as class and individual phytochemicals and, consequently, influences the health properties of the plant extracts differently, according to the matrix’s features. However, this traditional cooking method for the Capuchin monks’ salads proved to be a suitable method regarding the retention of phytochemicals and their health properties, unlike extended boiling, which resulted in compounds’ leaching into the blanching water.

Considering this, our results indicate that increased consumption of the investigated plants, even more if cooked according to the tradition, could provide a healthy food source in the modern diet by the recovery and enhancement of ancient ingredients.

In particular, *P. coronopus*, which resulted the most interesting plant species from this point of view, is an unconventional food plant, little known for its organoleptic and nutritional properties, which is until today little known and appreciated for its organoleptic and nutritional properties, that could be an excellent candidate to be exploited for new crops and new gastronomic uses.

## Figures and Tables

**Table 1:** Macro- and micromorphological features of the leaves of the selected species (PC, RA, CI and AD).

Species	Leaf Macromorphological Features	Leaf Micromorphological Features
*Plantago coronopus* L. (PC) Buck’s-horn plantain (Figure 1a)	Pubescent, toothed at the tip, slightly fleshy; narrow and pinnately lobed, arranged in a dense ascending rosette at the apex of a short stem [41].	Figure 2a–c *Epidermal cells*: rectangular with almost straight cell walls. *Stomata apparatus*: diacytic-type. *Trichomes*: two non-glandular types: bottle-like and larger, long stalked, multicellular trichomes [42]. Rarely, secretory trichomes could be observed [41].
*Rumex acetosa* L. (RA) Common sorrel (Figure 1b)	Large, ovate, hairless, fleshy; the lobes of basal leaves are pointed, and the petiole elongated; the stem leaves are almost stalkless [18].	Figure 2d–f *Epidermal cells*: irregularly shaped, with slightly undulating cell walls. *Stomata apparatus*: anisocytic and paracytic types. *Trichomes*: non-glandular trichomes lacking; glandular trichomes peltate, normally showing four-celled secretory heads [43].
*Cichorium intybus* L. (CI) Chicory (Figure 1c)	Hairy, arranged in an ascending rosette; oblong lanceolate, pinnate shape; basal leaves oblanceolate, toothed, with short petiole; cauline leaves smaller and sessile [24].	Figure 3a–c *Epidermal cells*: undulating cell walls. *Stomata apparatus*: anomocytic-type [44]. *Trichomes*: mutiseriate glandular trichomes on abaxial surface; multiseriate non-glandular trichomes with non-projecting cell apices on both surfaces [45].
*Artemisia dracunculus* L. (AD) Wild tarragon (Figure 1d)	Sessile, arranged alternately along the stem, with a sharp tip and entire leaf margins; lower leaves tripartite at the apex, the middle and upper leaves are lanceolate [30].	Figure 3d–f *Epidermal cells*: highly undulating cell walls. *Stomata apparatus*: anomocytic-type. *Trichomes*: stellate non-glandular trichomes and biseriate glandular trichomes with a subcuticular space filled with secondary compounds [46].

**Table 2:** Comparison between the phytochemical profiles of fresh (F) and cooked (C) leaf extracts of the traditional mixed-green salad of the Capuchin monks: *Plantago coronopus* L. (PC), *Rumex acetosa* L. (RA), *Cichorium intybus* L. (CI), and *Artemisia dracunculus* L. (AD). Results, which represent the average ± S.D. of three independent experiments in triplicate (*n* = 3), were expressed as mg of reference compound (gallic acid, quercetin, catechin and cyanidin for total phenols, flavonoids, vanillin index and proanthocyanidins, respectively) equivalents/100 g of dry extract (DE). Total chlorophyll and carbohydrates content were expressed as mg/g and g/100 g of fresh weight (FW), respectively.

Plant Extracts	Total Phenols	Flavonoids	Vanillin Index	Proanthocyanidins	Total Chlorophyll	Carbohydrates
PCF	1704.00 ± 93.22 ^a,d^	8594.25 ± 43.49 ^a,d^	446.96 ± 35.67 ^a,d^	1.27 ± 0.01 ^a,d^	0.93 ± 0.02 ^a^	0.60 ± 0.02 ^a,d^
RAF	644.71 ± 25.28 ^b,e^	2364.09 ± 7.01 ^b,e^	53.74 ± 3.88 ^b^	6.00 ± 0.25 ^b,e^	1.04 ± 0.03 ^b,e^	0.39 ± 0.01 ^b,e^
CIF	1025.83 ± 95.63 ^f^	3613.59 ± 68.05 ^c,f^	10.75 ± 0.67 ^c,f^	3.73 ± 0.17 ^c,f^	1.14 ± 0.02 ^f^	0.54 ± 0.02 ^c,f^
ADF	1170.29 ± 101.90	1567.33 ± 95.21 ^g^	134.36 ± 5.24 ^g^	0.07 ± 0.00 ^g^	1.13 ± 0.01	0.48 ± 0.01
PCC	0.88 ± 686.37 ^a^	0.83 ± 1002.87 ^a^	557.38 ± 10.34 ^a^	16.33 ± 0.58 ^a^	0.94 ± 0.02 ^a^	1.92 ± 0.03 ^a^
RAC	1202.31 ± 50.21 ^b^	2921.38 ± 83.13 ^b^	60.65 ± 3.94 ^b^	2.83 ± 0.05 ^b^	0.84 ± 0.01 ^b^	0.14 ± 0.00 ^b^
CIC	1476.96 ± 61.66 ^c^	6355.65 ± 93.30 ^c^	17.47 ± 0.49 ^c^	9.55 ± 0.12 ^c^	1.05 ± 0.03	0.21 ± 0.00 ^c^
ADC	1298.73 ± 84.36	8767.83 ± 430.86	685.52 ± 5.23	13.13 ± 0.24	1.09 ± 0.04	0.48 ± 0.01

^a^*p* < 0.05 vs. RAF or RAC, CIF or CIC, and ADF or ADC, between fresh and cooked extracts, respectively; ^b^ *p* < 0.05 vs. CIF or CIC, and ADF or ADC, between fresh and cooked extracts, respectively; ^c^ *p* < 0.05 vs. ADF or ADC, between fresh and cooked extracts, respectively; ^d^ *p* < 0.05 vs. PCC; ^e^ *p* < 0.05 vs. RAC; ^f^ *p* < 0.05 vs. CIC; ^g^ *p* < 0.05 vs. ADC.

**Table 3:** Comparison between the health properties of fresh (F) and cooked (C) leaf extracts of the traditional mixed-green salad of the Capuchin monks: *Plantago coronopus* L. (PC), *Rumex acetosa* L. (RC), *Cichorium intybus* L. (CI), and *Artemisia dracunculus* L. (AD). Results, which represent the average of three independent experiments in triplicate (*n* = 3), were expressed as half maximal inhibitory concentration (IC_50_, µg/mL) with confident limits (C.L.) at 95%.

Plant Extracts	TEAC	FRAP	ORAC	ICA	BDA	APA
PCF	230.20 (190.47–278.22) ^a,d^	244.38 (171.30–348.57) ^a,d^	2.41 (1.94–2.50) ^d,g^	22.66 (19.28–26.62) ^a,d^	970.22 (405.66–2330.12)	701.55 (350.55–1430.22)
RAF	1111.82 (831.15–1487.27) ^b,e^	1970.96 (1493.70–2600.70) ^b,e^	5.67 (4.60–6.98) ^b,e^	6.63 (5.79–7.60) ^c^	910.11 (762.33–1092.11)	1141.22 (891.99–1480.21) ^b^
CIF	510.96 (419.14–622.89) ^f^	841.48 (709.86–997.51) ^c,f^	1.89 (1.50–2.38) ^f^	7.80 (6.53–9.33)	1020.31 (862.05–1224.58)	540.34 (462.22–640.12)
ADF	331.66 (258.54–425.47)	546.40 (444.0–672.0)	1.36 (0.95–1.96)	9.85 (8.10–11.97)	980.08 (842.34–1162.66)	441.22 (380.05–510.11)
PCC	31.89 (27.06–37.59) ^a^	31.35 (26.51–37.09) ^a^	0.30 (0.25–0.37) ^a^	9.05 (7.49–10.95)	760.25 (650.22–890.27)	440.04 (190.10–992.90)
RAC	377.70 (296.34–481.39)	383.59 (322.90–455.69)	1.94 (1.58–2.39) ^f^	6.52 (5.76–7.38)	861.33 (731.22–1020.66)	871.22 (730.22–044.10) ^b^
CIC	273.80 (228.12–328.63)	396.67 (331.67–474.40)	1.10 (0.89–1.36)	7.64 (6.47–9.02)	970.02 (812.49–1151.08)	371.02 (142.04–960.07)
ADC	278.10 (220.15–353.57)	425.20 (343.0–527.0)	1.22 (0.93–1.60)	7.11 (6.06–8.35)	812.42 (692.77–951.55)	240.20 (212.33–298.99) ^c^
Standard	3.28 (2.44–3.89) ^h^	3.88 (1.62–5.78) ^h^	0.79 (0.39–1.65) ^i^	6.48 (5.22–7.68) ^l^	31.82 (26.58–38.10) ^h^	32.88 (25.22–39.13) ^h^

* Standard: trolox for TEAC, ORAC and FRAP assays, EDTA for iron-chelating activity (ICA), diclofenac sodium for BSA denaturation assay (BDA) and anti-protease activity (APA). ^a^ *p* < 0.05 vs. RAF or RAC, CIF or CIC, and ADF or ADC, between fresh and cooked extracts, respectively; ^b^ *p* < 0.05 vs. CIF or CIC, and ADF or ADC between fresh and cooked extracts, respectively; ^c^ *p* < 0.05 vs. ADF; ^d^ *p* < 0.05 vs. PCC; ^e^ *p* < 0.05 vs. RAC; ^f^ *p* < 0.05 vs. CIC; ^g^ *p* < 0.05 vs. RAF; ^h^ *p* < 0.05 vs. all extracts investigated; ^i^ *p* < 0.05 vs. PCF and RAF; ^l^ *p* < 0.05 vs. PCF and ADF.

## Data Availability

The data presented in this study are available on request from the corresponding author.
